# The potential association between common comorbidities and severity and mortality of coronavirus disease 2019: A pooled analysis

**DOI:** 10.1002/clc.23465

**Published:** 2020-10-07

**Authors:** Liman Luo, Menglu Fu, Yuanyuan Li, Shuiqing Hu, Jinlan Luo, Zhihui Chen, Jing Yu, Wenhua Li, Ruolan Dong, Yan Yang, Ling Tu, Xizhen Xu

**Affiliations:** ^1^ Department of Geriatric Medicine, Tongji Hospital, Tongji Medical College Huazhong University of Science and Technology Wuhan Hubei China; ^2^ Division of Cardiology and Department of Internal Medicine, Tongji Hospital Tongji Medical College, Huazhong University of Science and Technology Wuhan Hubei China; ^3^ Institute of Integrated Traditional Chinese and Western Medicine, Tongji Hospital Tongji Medical College, Huazhong University of Science and Technology Wuhan China; ^4^ Division of Endocrinology and Department of Internal Medicine, Tongji Hospital Tongji Medical College, Huazhong University of Science and Technology Wuhan China; ^5^ Hubei Key Laboratory of Genetics and Molecular Mechanisms of Cardiological Disorders Tongji Medical College, Huazhong University of Science and Technology Wuhan Hubei China

**Keywords:** cardiac injury, comorbidities, COVID‐19, meta‐analysis, mortality, severity

## Abstract

**Backgroud:**

The association between underlying comorbidities and cardiac injury and the prognosis in coronavirus disease 2019 (COVID‐19) patients was assessed in this study.

**Hypothesis:**

The underlying comorbidities and cardiac injury may be associated with the prognosis in COVID‐19 patients.

**Methods:**

A systematic search was conducted in PubMed, EMBASE, Web of science, and The Cochrane library from December 2019 to July 2020. The odds ratio (OR) and 95% confidence intervals (95% CI) were used to estimate the probability of comorbidities and cardiac injury in COVID‐19 patients with or without severe type, or in survivors vs nonsurvivors of COVID‐19 patients.

**Results:**

A total of 124 studies were included in this analysis. A higher risk for severity was observed in COVID‐19 patients with comorbidities. The pooled result in patients with hypertension (OR 2.57, 95% CI: 2.12‐3.11), diabetes (OR 2.54, 95% CI: 1.89‐3.41), cardiovascular diseases (OR 3.86, 95% CI: 2.70‐5.52), chronic obstractive pulmonary disease (OR 2.71, 95% CI: 1.98‐3.70), chronic kidney disease (OR 2.20, 95% CI: 1.27‐3.80), and cancer (OR 2.42, 95% CI: 1.81‐3.22) respectively. All the comorbidities presented a higher risk of mortality. Moreover, the prevalence of acute cardiac injury is higher in severe group than in nonsevere group, and acute cardiac injury is associated with an increased risk for in‐hospital mortality.

**Conclusion:**

Comorbidities and acute cardiac injury are closely associated with poor prognosis in COVID‐19 patients. It is necessary to continuously monitor related clinical indicators of organs injury and concern comorbidities in COVID‐19 patients.

## 1 INTRODUCTION

Coronavirus disease 2019 (COVID‐19) has emerged as a global pandemic and a public health event of widespread concern.^1^, ^2^, ^3^, ^4^, ^5^ To date, more than 3.4 million individuals worldwide with confirmed COVID‐19, of whom more than 200 000 have lost their lives. The higher incidence of comorbidities in COVID‐19 patients, including hypertension, diabetes and cardiovascular disease et al, was reported in recent retrospective studies. Cardiovascular metabolic comorbidities may be a risk factor for poor prognosis. In addition, COVID‐19 patients have different degrees of myocardial damage in addition to respiratory symptoms, especially in critically ill patients.

Huang et al.^6^ first reported that 32% of the cases had comorbidities, 8% developed myocardial injury, and the mortality rate as high as 15% in 41 confirmed patients. The study of Yang et al.^7^ indicated that elderly critically ill patients (> 65 years old) with comorbidities and acute respiratory distress syndrome (ARDS) are at higher risk of death. In addition, Guo T et al^8^ and Shi et al.^9^ reported that COVID‐19 patients with myocardial injury had a significantly higher mortality rate than patients without myocardial injury. Given the worldwide pandemic of this infectious disease, it is necessary to identify the risk factors associated with increased risk of in‐hospital mortality in COVID‐19 patients. Although, some clinical case series studies demonstrated that comorbidities including hypertension, diabetes, and cardiovascular diseases (CVD), chronic obstractive pulmonary disease (COPD), chronic kidney disease (CKD), and cancer may be predictors for the poor prognosis in COVID‐19 patients, the number of enrolled patients was limited, and potential confounding factors were not excluded, so it still needs to be further confirmed. In this study, we conducted a systematic review of available studies to assess the association between underlying comorbidities and acute cardiac injury and the severity or the prognosis in COVID‐19 patients.

## 2 METHODS

### 2.1 Search strategy and study selection

We conducted systematic retrieval in PubMed, EMBASE (by Ovidsp), Web of Science and The Cochrane Library from December 2019 to July 2020. The free keywords included “comorbidities”, “hypertension”, “diabetes”, “cardiovascular disease”, “cardiac injury”, “chronic obstractive pulmonary disease”, “chronic kidney disease”, “cancer”, “novel coronavirus pneumonia”, “COVID‐19”, “2019‐nCoV” and “clinical characteristics”. Additionally, we checked the references of each cited manuscript to identify other possibly eligible studies. This pooled analysis was conducted and reported in compliance with the preferred reporting items for systematic reviews and meta‐analyses (PRISMA) guidelines.^10^

Eligible studies should be written in English, and describe the relationship between age, gender, comorbidities and the prognosis of adult COVID‐19 patients. The number of enrolled patients is more than ten. Case reports, reviews, letters, family‐based studies, nonhuman studies, studies without adequate information, studies written not in English, studies focused only on children or pregnant women, and patients not stratified with the degree of severity or survivors were excluded. The inclusion of each study was determined by two researchers. Disagreements were resolved through a consensus.

### 2.2 Data extraction

Two reviewers independently extracted data from the included studies. Discrepancies were resolved by consensus between the two reviewers. Variables included authors, sample size, age, gender, relevant data on comorbidity of severe and nonsevere, and of survivors and nonsurvivors (including hypertension, diabetes, CVD, COPD, CKD, and cardiac injury). Cardiac injury was defined by elevation of Troponin I/T. The New castle Ottawa scale (NOS) was followed to assess the quality of studies.^11^ The primary outcome was to explore the association between the comorbidities or acute cardiac injury and severity or mortality in confirmed COVID‐19 patients.

### 2.3 Statistical analysis

Pooled analysis was performed using STATA software (version 14.0). The odds ratio (OR) and 95% confidence intervals (95% CI) were used to estimate the probability of comorbidities or cardiac injury in COVID‐19 patients with or without severe type, or in survivors vs nonsurvivors of COVID‐19 patients. Magnitude of heterogeneity was calculated using the I^2^ statistic: 25%, 50%, and 75% representing low, medium, and high heterogeneity, respectively. Due to the heterogeneity between studies, a random effect model was performed to estimating the average effect.^12^ In order to assess the impact of age (mean age or median age) and sex (percentage of males), univariable meta‐regression models were performed. Publication bias was evaluated by the Bgger's test, with *P* > .05 indicated no evidence of publication bias.^13^

## 3 RESULTS

### 3.1 Search results and study characteristics

**FIGURE 1:**
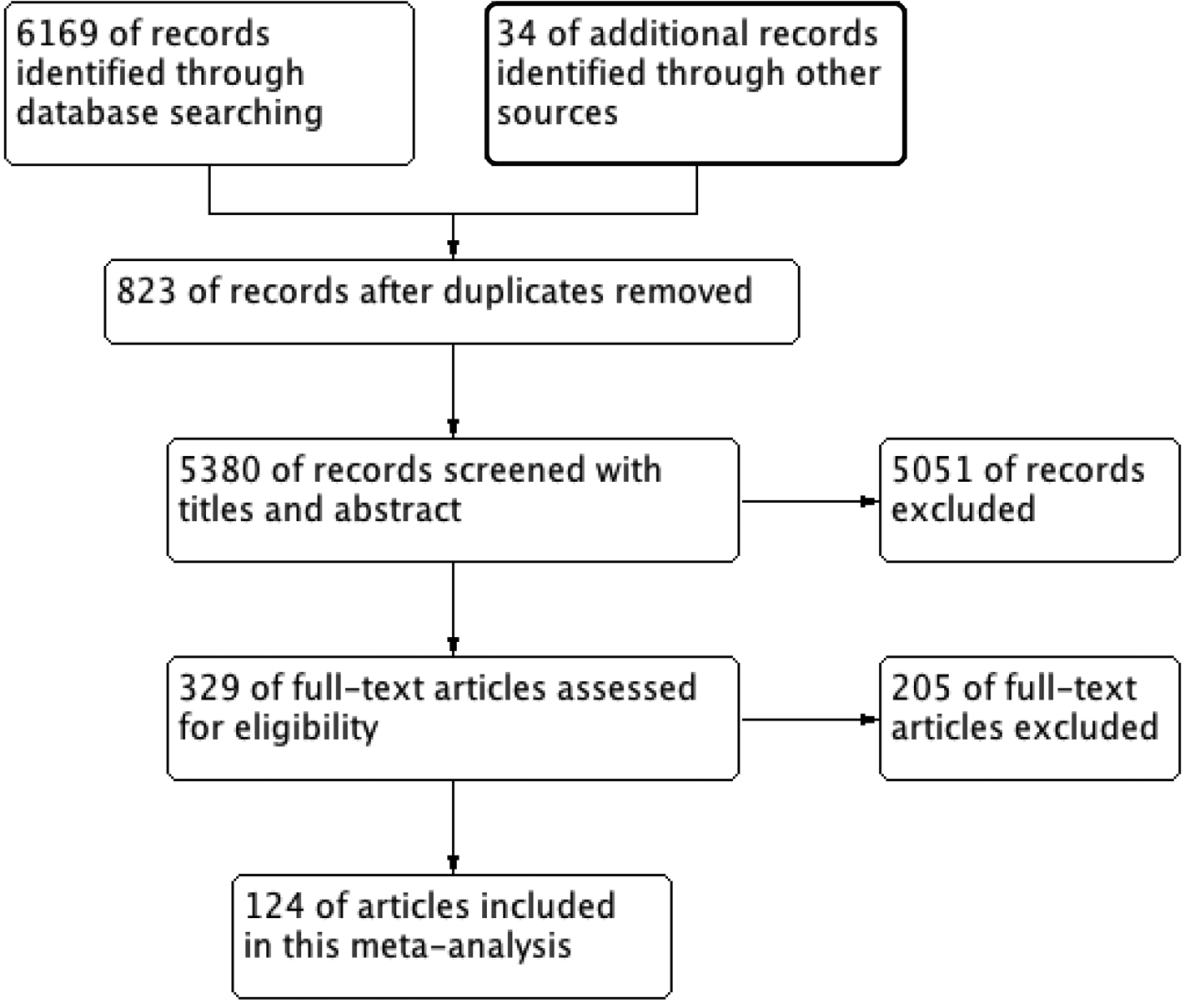
The flow of studies literature search and selection of studies

Initial database search identified 6196 studies and 34 additional records through reference and citation searches (Figure 1). Overall, 329 of them with full‐text were reviewed for eligibility, of which 124 studies were included in this analysis.^7^, ^8^, ^9^, ^14^, ^15^, ^16^, ^17^, ^18^, ^19^, ^20^, ^21^, ^22^, ^23^, ^24^, ^25^, ^26^, ^27^, ^28^, ^29^, ^30^, ^31^, ^32^, ^33^, ^34^, ^35^, ^36^, ^37^, ^38^, ^39^, ^40^, ^41^, ^42^, ^43^, ^44^, ^45^, ^46^, ^47^, ^48^, ^49^, ^50^, ^51^, ^52^, ^53^, ^54^, ^55^, ^56^, ^57^, ^58^, ^59^, ^60^, ^61^, ^62^, ^63^, ^64^, ^65^, ^66^, ^67^, ^68^, ^69^, ^70^, ^71^, ^72^, ^73^, ^74^, ^75^, ^76^, ^77^, ^78^, ^79^, ^80^, ^81^, ^82^, ^83^, ^84^, ^85^, ^86^, ^87^, ^88^, ^89^, ^90^, ^91^, ^92^, ^93^, ^94^, ^95^, ^96^, ^97^, ^98^, ^99^, ^100^, ^101^, ^102^, ^103^, ^104^, ^105^, ^106^, ^107^, ^108^, ^109^, ^110^, ^111^, ^112^, ^113^, ^114^, ^115^, ^116^, ^117^, ^118^, ^119^, ^120^, ^121^, ^122^, ^123^, ^124^, ^125^, ^126^, ^127^, ^128^, ^129^, ^130^, ^131^, ^132^, ^133^, ^134^ The NOS scores ranged between 5 and 8. The key characteristics of included studies were presented in Table S1. The majority of included studies were based in Asia, the minority was from the USA, Italy, Spain and other countries. With most studies including more males than females, and the mean age varied from a median of 40 to 84 years of age. 58 studies compared the incidence of hypertension or diabetes in severe vs nonsevere cases with COVID‐19 and 29 studies compared the prevalence of CVD. Eleven studies reported the association between acute cardiac injury and the severity of COVID‐19. Sixty three documents reported the incidence of cardiovascular metabolic diseases in nonsurvivors vs survivors.

### 3.2 Meta‐analysis

**FIGURE 2:**
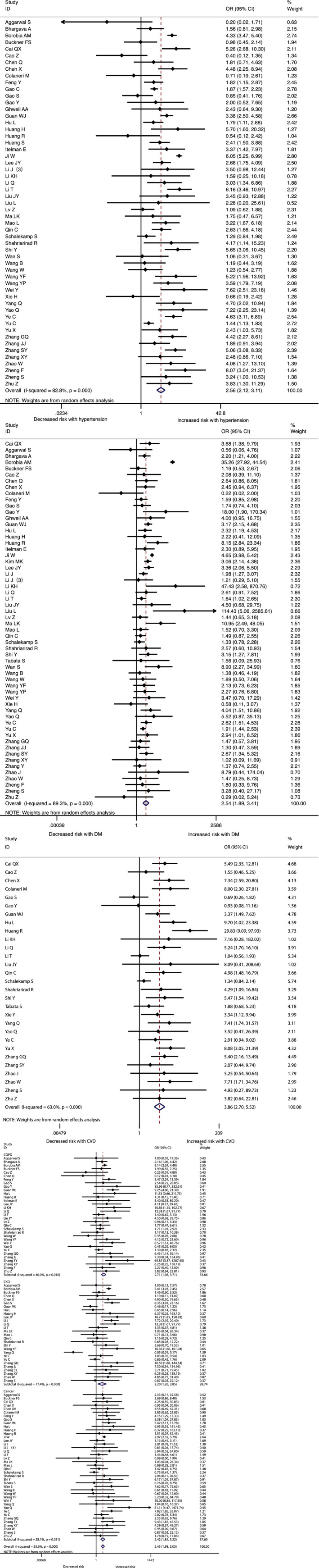
The association of comorbidities (hypertension, diabetes, and cardiovascular diseases(CVD), chronic obstractive pulmonary disease(COPD), chronic kidney disease(CKD), and cancer)with COVID‐19 severity

**FIGURE 3:**
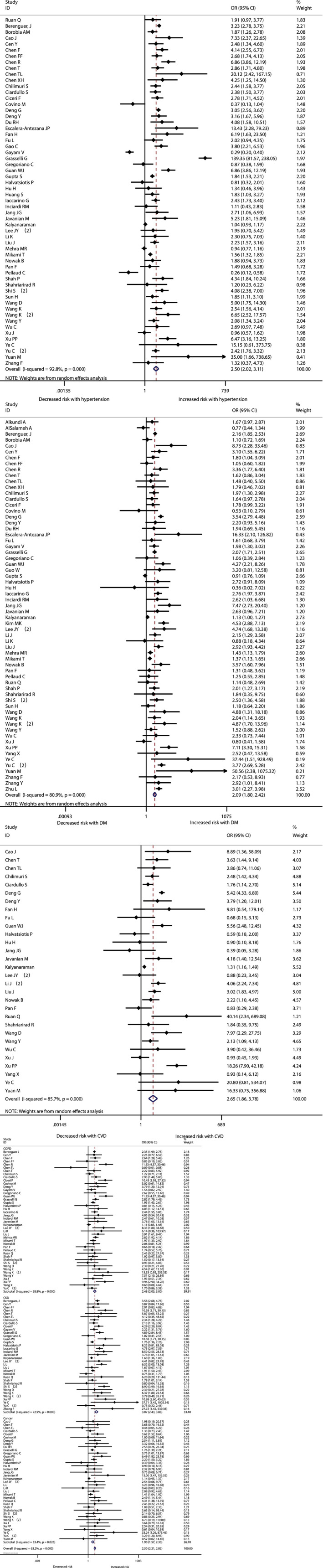
The association of comorbidities (hypertension, diabetes, and cardiovascular diseases(CVD), chronic obstractive pulmonary disease(COPD), chronic kidney disease(CKD), and cancer) with COVID‐19 mortality

**FIGURE 4:**
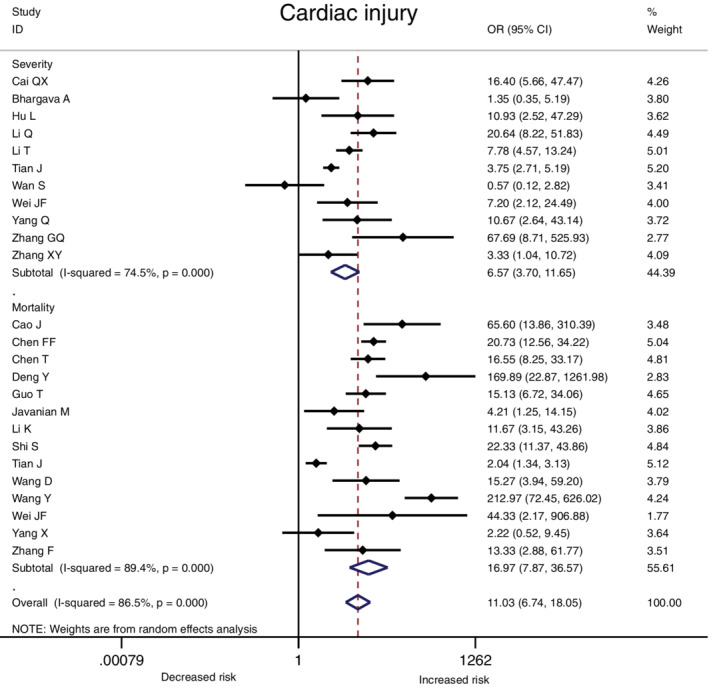
The association of cardiac injury with COVID‐19 severity and mortality

The association between comorbidities and disease severity were presented in Figure 2. A heterogeneity between the studies varied from moderate to high. A higher risk for severity was observed in COVID‐19 patients with hypertension, diabetes, CVD, COPD, CKD or cancer, and the pooled result was (OR 2.57, 95% CI: 2.12‐3.11), (OR 2.54, 95% CI: 1.89‐3.41), (OR 3.86, 95% CI: 2.70‐5.52), (OR 2.71, 95% CI: 1.98‐3.70), (OR 2.20, 95% CI: 1.27‐3.80) and (OR 2.42, 95% CI: 1.81‐3.22) respectively (Figure 2, Table S2). At the same time, the risk for in‐hospital mortality was significantly increased in COVID‐19 patients with hypertension (OR 2.50, 95% CI: 2.02‐3.11), diabetes (OR 2.09, 95% CI: 1.80‐2.42), CVD (OR 2.65, 95% CI: 1.87‐3.77), COPD (OR 2.48, 95% CI: 2.05‐3.00), CKD (OR 3.07, 95% CI: 2.43‐3.88) or cancer (OR 1.90, 95% CI: 1.57‐2.30) (Figure 3, Table S2). Moreover, it is observed that the prevalence of acute cardiac injury is higher in severe group than in nonsevere group (OR:6.57; 95% CI 3.70‐11.65), and acute cardiac injury is associated with an increased risk for mortality (OR:16.96; 95% CI 7.89‐36.44) (Figure 4).

### 3.3 Meta‐regression analysis

The results of univariable meta‐regression analyses showed the impact of age and sex on the association between comorbidities or acute cardiac injury and the prognosis (severity and mortality) in COVID‐19 patients. There was a clearer effect of increasing age on the association between hypertesion and diabetes and severity of COVID‐19 (sFig1, sFig2). Conversely, there is no significant association between the proportion of males with the risk of severity or mortality. No obvious evidence of publication bias existed (Table S2).

## 4 DISCUSSION

The pandemic of COVID‐19 poses a huge challenge to countries all over the world. SARS‐CoV‐2 infection is more serious among individuals with immune deficiency and comorbidities. Reliable population epidemiology, clinical characteristics, and laboratory parameters can help distinguish high‐risk COVID‐19 patients and enable more active management of hospitalized or critically ill patients. At present, some laboratory indicators that may predict the deterioration of COVID‐19 have been identified, including leukocytosis, lymphopenia, thrombocytopenia, and cardiac injury biomarkers, elevated inflammatory cytokines. Notably, early studies reported some clinical indicators such as age, gender, as well as existing hypertension, diabetes and CVD can predict the prognosis of COVID‐19. CVD have the highest prevalence among potential patients at higher risk for severe acute respiratory syndrome coronavirus 2 (SARS‐CoV‐2) infection.^135^ Comorbidities such as hypertension and diabetes are recognized as poor prognostic factors for ARDS and SIRS.^136^, ^137^ However, the relationship between these comorbidities and COVID‐19 severity or poor survival outcomes remains unclear.

This pooled analysis is based on data from 124 studies with confirmed COVID‐19. In this study, it is observed that the comorbidities makes COVID‐19 patients more likely to develop severe clinical type and increase the risk for in‐hospital death. Comorbidities may be a risk factor for critically ill patients with poor prognosis. Another important finding is that acute cardiac injury increased the risk of severity and in‐hospital death for patients with COVID‐19. According to the current research data, some patients with COVID‐19 suffered acute cardiac injury, and the incidence of acute cardiac injury is much higher in severe cases.^6^, ^21^, ^24^ However, the pathogenesis of acute cardiac injury associated with COVID‐19 is still needs further investigation. The unique and significant affinity of SARS‐CoV‐2 for the host ACE2 receptor increases the possibility of direct infection of vascular endothelium and myocardium. Meanwhile, hypoxemia and cytokine storm may also be an important cause of acute cardiac injury.^6^ Therefore, it is necessary to monitor acute cardiac injury markers and cardiac function during hospitalization, and pay more attention to heart damage related to SARS‐CoV‐2 infection in the course of disease treatment, and take more active treatment for patients with acute cardiac injury.

A recent study on influenza showed that patients with cardiovascular disease and hypertension have a higher risk of death than those without comorbidities.^138^ Previous studies in patients with Middle East Respiratory Syndrome Coronavirus (MERS‐CoV) found that comorbidities were significantly associated with poor prognosis.^139^ Recently, meta‐analysis have evaluated the impact of comorbidities in the population of COVID‐19, and the results are consistent with this review,^140^, ^141^, ^142^, ^143^but with fewer the included studies. This pooled results are based on more data and included studies from many countries including China, the United States, Italy and so on. However, the local policy of COVID‐19 research and the time of patient enrollment is different, and some studies only included severely ill or elderly patients. Over time, people's attention to the epidemic, the implementation of related policies, and improvement of treatment plans may reduce the overall severity and mortality of COVID‐19. In addition, there are reports that some controversial studies of COVID‐19 patients have been withdrawn after the author failed to demonstrate the reliability of the data. This review included retrospective observational studies, which are prone to selection and recall bias in the collection and processing of data, and the definition of outcome may be different in each study. In order to make our results as accurate as possible, we removed studies that clearly had overlapping cohorts because they specified the same hospital and time period. However, we cannot be sure that the data among all studies does not overlap. The actual prevalence of comorbidities in COVID‐19 patients and their impact on prognosis remain unknown.

In summary, we updates the evidence on the association between comorbidities or cardiac injury and COVID‐19. The pooled result support that patients with comorbidities may increase the severity of SARS‐CoV‐2 infection, and may also greatly affect the survival outcome of COVID‐19 patients. The successful treatment of severe or critical cases is the key to reduce in‐hospital mortality. Prevention and intervention measures for these patients should be strengthened, and patients with underlying chronic diseases are strongly recommended to avoid any close contact with others in the community, especially in endemic areas. It is necessary to continuously monitor blood pressure, blood glucose, and related clinical indicators of organs injury in COVID‐19 patients with comorbidities.

Indeed, there are some limitations in this study. First, the baseline characteristics of included population in various studies may bias the results. Second, patients with one or more comorbidities and different clinical treatment strategies will lead to different survival outcomes. In addition, although we collect as much complete and reliable data as possible, most of the studies included are from China since it is the main focus of the pandemic rise. The actual prevalence and mortality rates may vary in different countries. Finally, we evaluated publication bias through Bgger regression test, but currently these common statistical methods (such as funnel plot, Bgger test and Egger test) are not considered to be useful evaluation tools. The influence of publication bias cannot be completely ruled out. Prospective randomized control trials are warranted to further confirm the conclusions in this study.

## 5 CONCLUSION

Comorbidities and acute cardiac injury are closely associated with poor prognosis in COVID‐19 patients. It is necessary to continuously monitor related clinical indicators of organs injury and concern comorbidities in COVID‐19 patients.

## CONFLICT OF INTEREST

The authors declare they have no conflict of interest.

## Supporting information


**Figure S1** A Univariable linear meta‐regression analyses of the association of either age (panels A and C) or sex (panels B and D) the hypertention‐related risk of COVID‐19 severity or mortality.
**sFig 2.** A Univariable linear meta‐regression analyses of the association of either age (panels A and C) or sex (panels B and D) the diabetes‐related risk of COVID‐19 severity or mortality.
**sFig 3.** A Univariable linear meta‐regression analyses of the association of either age (panels A and C) or sex (panels B and D) the cardiovascular disease‐related risk of COVID‐19 severity or mortality.
**sFig 4.** A Univariable linear meta‐regression analyses of the association of either age (panels A and C) or sex (panels B and D) the COPD‐related risk of COVID‐19 severity or mortality.
**sFig 5.** A Univariable linear meta‐regression analyses of the association of either age (panels A and C) or sex (panels B and D) the CKD‐related risk of COVID‐19 severity or mortality.
**sFig 6.** A Univariable linear meta‐regression analyses of the association of either age (panels A and C) or sex (panels B and D) the cancer‐related risk of COVID‐19 severity or mortality.
**sFig 7.** A Univariable linear meta‐regression analyses of the association of either age (panels A and C) or sex (panels B and D) the cardiac injury ‐related risk of COVID‐19 severity or mortality.

**Table S1** Baseline characteristics of studies included in the systematic review.
**Table S2**: The pooled outcome in patients with comorbidities or cardiac injury.

## Data Availability

The data supporting this systematic review are from previously reported studies and datasets, which have been cited.
