# Supplementary material for: The potential association between common comorbidities and severity and mortality of coronavirus disease 2019: A pooled analysis
Source: Clin Cardiol. 2020 Oct 7;43(12):1478–93. doi: 10.1002/clc.23465 (PMC7675427; doi:10.1002/clc.23465)
Supplement: Supplementary file 1 — Figure S1 A Univariable linear meta‐regression analyses of the association of either age (panels A and C) or sex (panels B and D) the hypertention‐related risk of COVID‐19 severity or mortality. sFig 2. A Univariable linear meta‐regression analyses of the association of either age (panels A and C) or sex (panels B and D) the diabetes‐related risk of COVID‐19 severity or mortality. sFig 3. A Univariable linear meta‐regression analyses of the association of either age (panels A and C) or sex (panels B and D) the cardiovascular disease‐related risk of COVID‐19 severity or mortality. sFig 4. A Univariable linear meta‐regression analyses of the association of either age (panels A and C) or sex (panels B and D) the COPD‐related risk of COVID‐19 severity or mortality. sFig 5. A Univariable linear meta‐regression analyses of the association of either age (panels A and C) or sex (panels B and D) the CKD‐related risk of COVID‐19 severity or mortality. sFig 6. A Univariable linear meta‐regression analyses of the association of either age (panels A and C) or sex (panels B and D) the cancer‐related risk of COVID‐19 severity or mortality. sFig 7. A Univariable linear meta‐regression analyses of the association of either age (panels A and C) or sex (panels B and D) the cardiac injury ‐related risk of COVID‐19 severity or mortality. [file CLC-43-1478-s001.pdf]

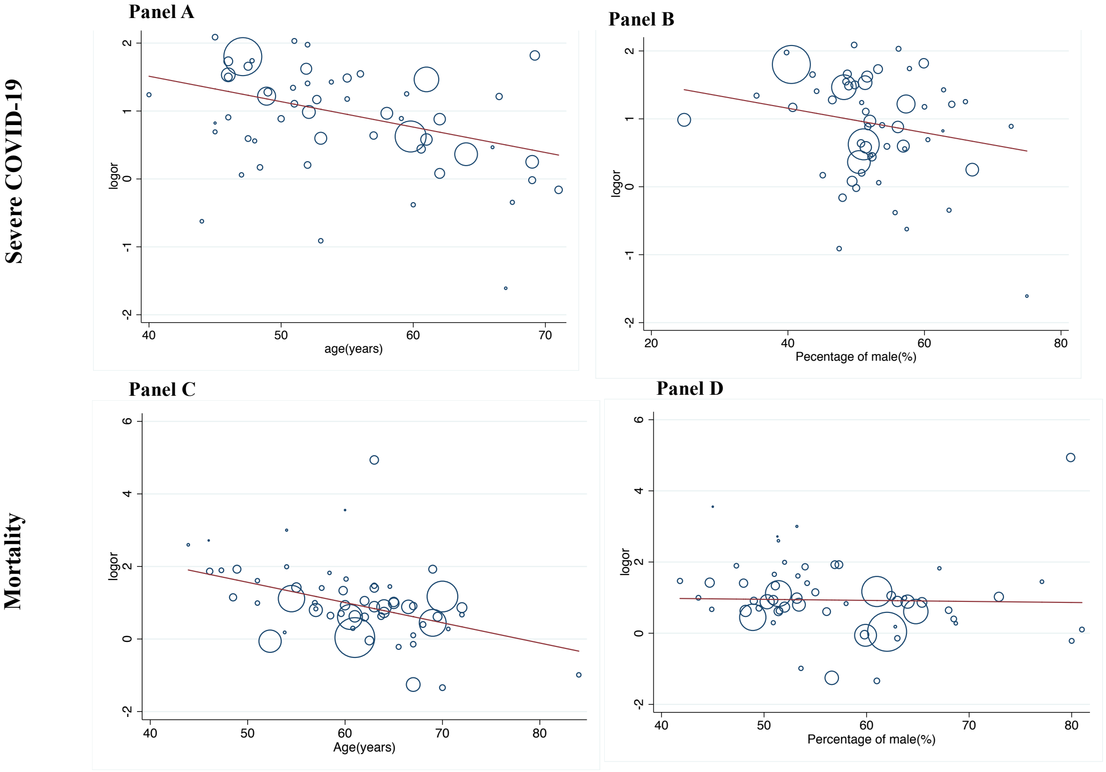

sFig 1: A Univariable linear meta-regression analyses of the association of either age (panels A and C) or sex (panels B and D) the hypertention-related risk of COVID-19 severity or mortality.

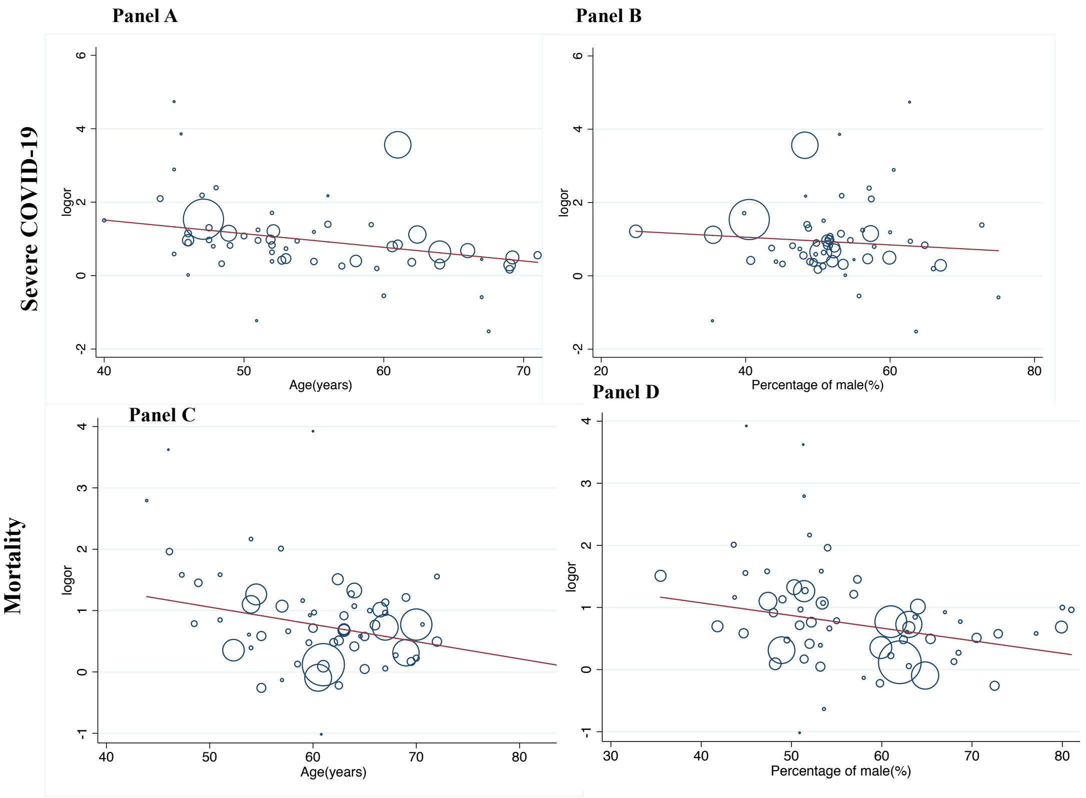

sFig 2: A Univariable linear meta-regression analyses of the association of either age (panels A and C) or sex (panels B and D) the diabetes-related risk of COVID-19 severity or mortality.

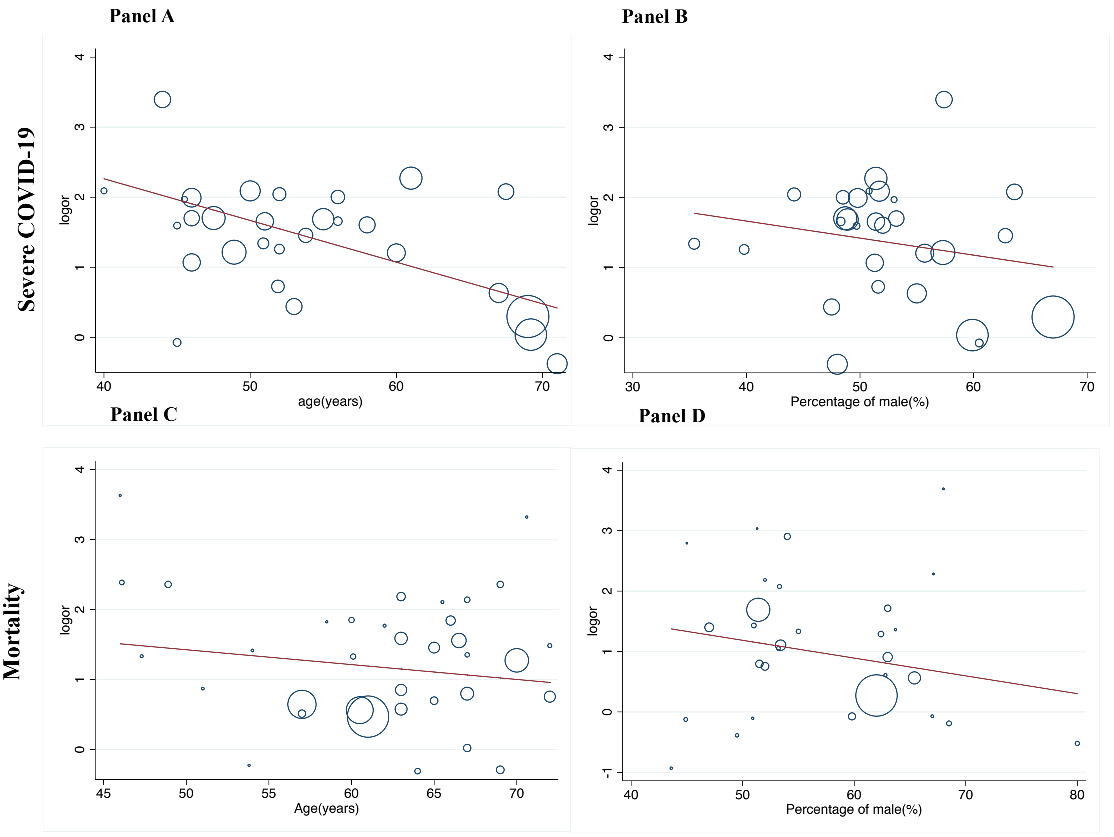

sFig 3: A Univariable linear meta-regression analyses of the association of either age (panels A and C) or sex (panels B and D) the cardiovascular disease-related risk of COVID-19 severity or mortality.

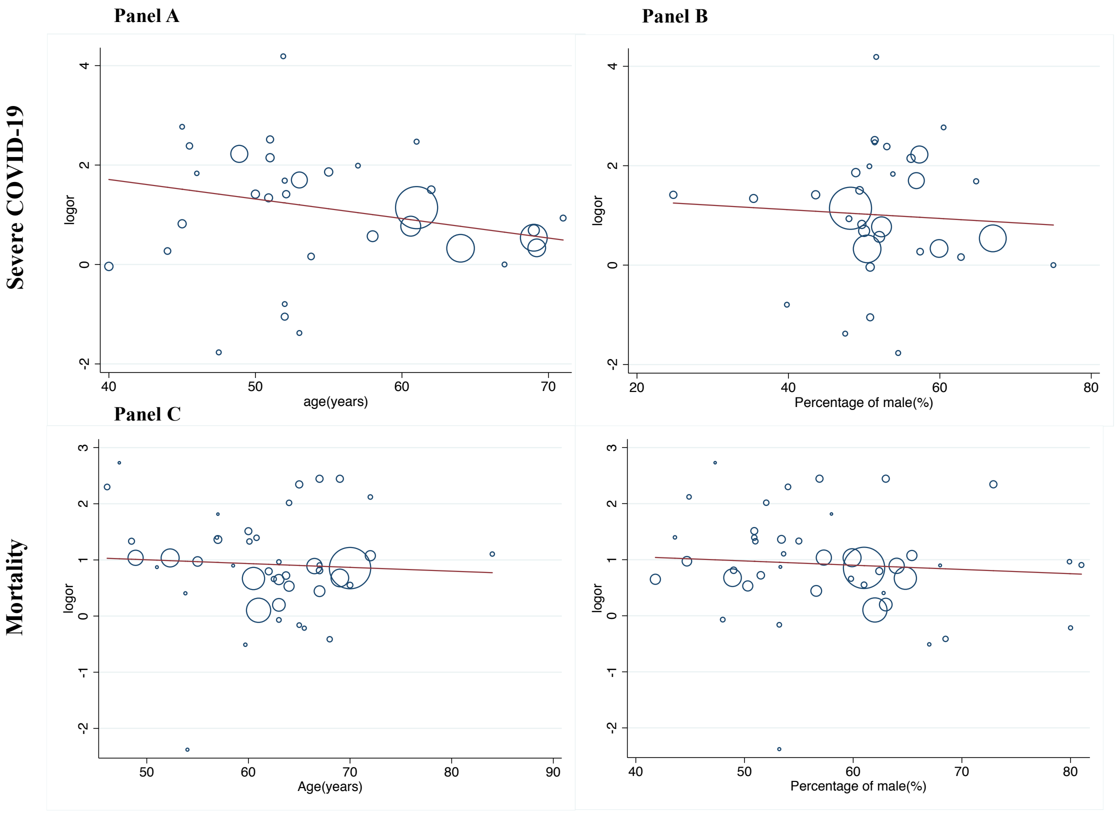

sFig 4: A Univariable linear meta-regression analyses of the association of either age (panels A and C) or sex (panels B and D) the COPD-related risk of COVID-19 severity or mortality.

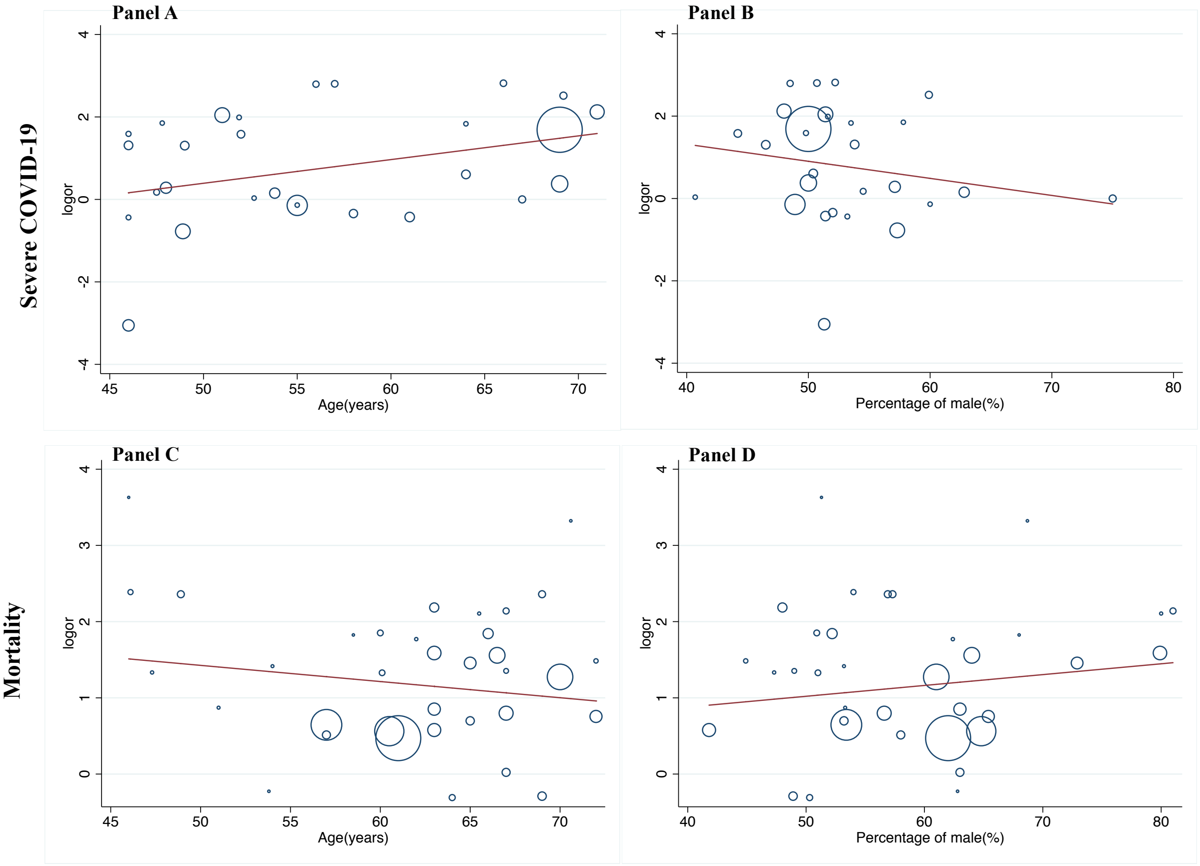

sFig 5: A Univariable linear meta-regression analyses of the association of either age (panels A and C) or sex (panels B and D) the CKD-related risk of COVID-19 severity or mortality.

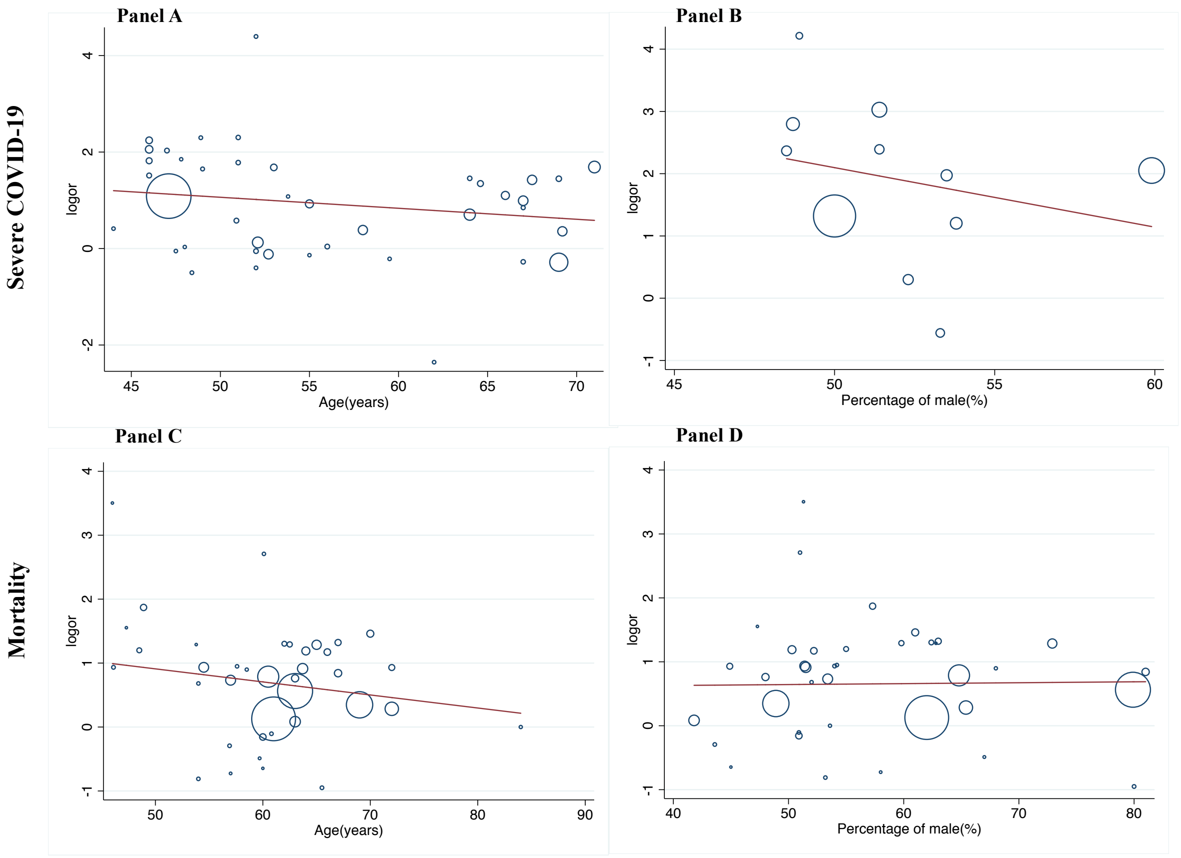

sFig 6: A Univariable linear meta-regression analyses of the association of either age (panels A and C) or sex (panels B and D) the cancer-related risk of COVID-19 severity or mortality.

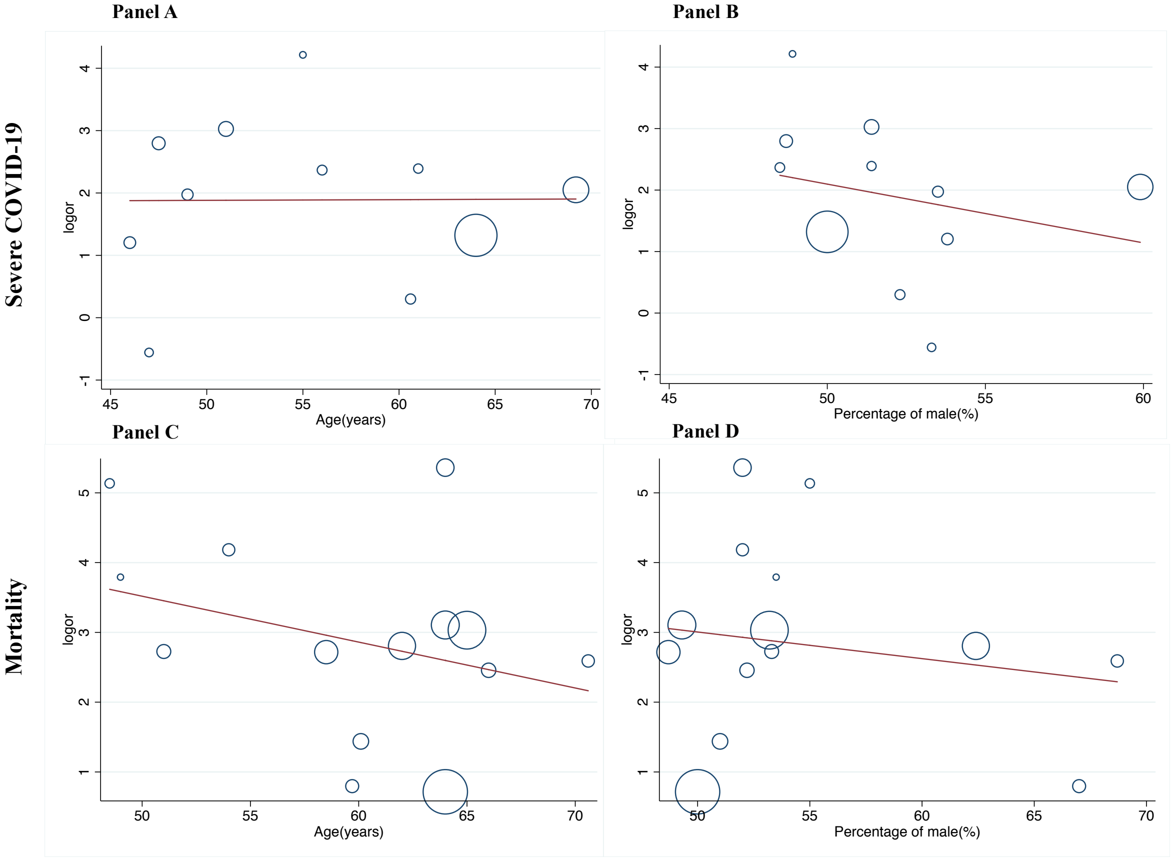

sFig 7: A Univariable linear meta-regression analyses of the association of either age (panels A and C) or sex (panels B and D) the cardiac injury -related risk of COVID-19 severity or mortality.
