# Supplementary material for: The potential association between common comorbidities and severity and mortality of coronavirus disease 2019: A pooled analysis
Source: Clin Cardiol. 2020 Oct 7;43(12):1478–93. doi: 10.1002/clc.23465 (PMC7675427; doi:10.1002/clc.23465)
Supplement: Supplementary file 2 — Table S1 Baseline characteristics of studies included in the systematic review. Table S2: The pooled outcome in patients with comorbidities or cardiac injury. [file CLC-43-1478-s002.pdf]

sTable 1. Baseline characteristics of studies included in the systematic review.

| Study | Country | All | Male (%) | Severe(%) | Death (%) | average age | HTN(%) | DM (%) | CVD (%) | Cardiac injury (%) | COPD (%) | CKD (%) | Cancer (%) |
|---|---|---|---|---|---|---|---|---|---|---|---|---|---|
| Aggarwal S | America | 16 | 75.0 | 50.0 | NA | 67.0 | 57.0 | 31.0 | NA | NA | 13.0 | 38.0 | 19.0 |
| Alkundi A | United Kingdom | 232 | 62.5 | NA | 38.4 | 70.5 | 14.7 | 37.5 | NA | NA | 7.3 | NA | 3.0 |
| AlSalameh A | France | 433 | 55.0 | 18.5 | 20.3 | 72.5 | 59.1 | 26.6 | 45.7 | 8.8 | 9.0 | 15.2 | 16.6 |
| Berenguer, J | Spain | 4035 | 61.0 | NA | 28.0 | 70.0 | 51.2 | 21.8 | NA | NA | 17.9 | NA | NA |
| Bhargava A | America | 197 | 52.3 | 37.6 | NA | 60.6 | 70.1 | 37.1 | NA | 4.6 | 19.3 | NA | NA |
| Borobia AM | Spain | 2226 | 48.2 | NA | 20.7 | 61.0 | 41.3 | 17.1 | NA | NA | 6.9 | 7.8 | NA |
| Buckner FS | America | 105 | 50.0 | 48.6 | 33.0 | 69.0 | 59.0 | 33.0 | 38.0 | 19.0 | 10.0 | 26.0 | 15.0 |
| Cai QX | China | 298 | 48.7 | 19.5 | 1.0 | 47.5 | 15.8 | 6.0 | 8.4 | 6.7 | NA | NA | 1.3 |
| Cao J | China | 102 | 52.0 | 17.6 | 16.7 | 54.0 | 27.5 | 10.8 | 4.9 | 14.7 | NA | 3.9 | NA |
| Cao Z | China | 80 | 47.5 | 33.8 | NA | 53.0 | 25.0 | 7.5 | 12.5 | NA | 6.3 | NA | NA |
| Cen Y | China | 1007 | 49.0 | 24.2 | 4.3 | 67.0 | 26.8 | 11.8 | NA | NA | 4.6 | 1.4 | NA |
| Chen F | China | 660 | 44.7 | NA | 12.4 | 55.0 | 34.8 | 17.3 | NA | NA | 6.5 | NA | NA |
| Chen FF | China | 681 | 53.2 | NA | 15.3 | 65.0 | 43.0 | 16.7 | NA | 20.4 | 2.2 | 4.0 | NA |
| Chen Q | China | 145 | 54.5 | 29.6 | NA | 47.5 | 15.2 | 9.7 | NA | NA | 4.1 | 2.1 | NA |
| Chen R | China | 1590 | 56.9 | NA | 3.1 | 69.0 | 16.9 | 8.2 | NA | NA | 1.5 | 1.3 | NA |
| Chen T | China | 274 | 62.4 | NA | 41.2 | 62.0 | 33.9 | 17.2 | 8.4 | 44.0 | 6.6 | 1.5 | 2.6 |
| Chen TL | China | 203 | 53.2 | 52.7 | NA | 54.0 | 21.2 | 7.9 | 7.9 | NA | 3.9 | 3.9 | 3.4 |
| Chen X | China | 291 | 49.8 | 17.2 | 0.7 | 46.0 | 13.4 | 7.6 | 4.1 | NA | 3.4 | 0.7 | 0.7 |
| Chen XH | China | 48 | 77.1 | 56.3 | 6.3 | 64.6 | 49.7 | 25.0 | NA | NA | NA | NA | 12.5 |
| Chilimuri S | America | 375 | 63.0 | NA | 42.7 | 63.0 | 60.0 | 47.0 | 17.0 | NA | 17.0 | 14.0 | NA |
| Ciardullo S | Italy | 373 | 65.4 | NA | 38.1 | 72.0 | 64.8 | 18.5 | 37.7 | NA | 10.6 | 12.9 | 13.6 |
| Ciceri F | Italy | 410 | 72.9 | NA | 23.2 | 65.0 | 49.9 | 15.0 | NA | NA | 5.4 | 11.8 | 5.4 |
| Colaneri M | Italy | 44 | 63.6 | 38.6 | 4.5 | 67.5 | 34.1 | 15.9 | 25.0 | NA | NA | NA | 13.6 |
| Covino M | Italy | 69 | 53.6 | 79.7 | 33.3 | 84.0 | 59.4 | 13.0 | NA | NA | 10.1 | NA | 4.3 |
| Deng G | China | 44672 | 51.4 | 18.5 | 2.3 | NA | 12.8 | 5.3 | 4.2 | 2.4 | NA | NA | 0.5 |
| Deng Y | China | 225 | 55.0 | NA | 48.4 | 48.5 | 25.8 | 11.6 | 7.6 | 29.3 | 7.0 | NA | 3.6 |
| Du RH | China | 179 | 54.2 | NA | 11.3 | 57.6 | 32.4 | 18.4 | 16.2 | NA | NA | 2.2 | 2.2 |
| Escalera-Antezana JP | Bolivia | 107 | 51.4 | NA | NA | 43.9 | 9.4 | 4.7 | NA | NA | NA | NA | NA |
| Fan H | China | 73 | 67.1 | NA | 64.4 | 58.4 | 32.9 | 16.4 | 9.6 | NA | NA | NA | NA |
| Feng Y | China | 476 | 56.9 | 26.1 | 8.0 | 53.0 | 23.7 | 10.3 | 8.0 | NA | 4.6 | NA | 2.5 |

| | | | | | | | | | | | | | |
|---|---|---|---|---|---|---|---|---|---|---|---|---|---|
| Fu L | China | 200 | 49.5 | NA | 16.0 | 59.6 | 50.5 | 68.5 | 8.0 | NA | 4.0 | NA | NA |
| Gao C | China | 2877 | 51.1 | 25.9 | 1.9 | NA | 29.5 | 13.5 | NA | 0.4 | 1.1 | 1.0 | 1.7 |
| Gao S | China | 210 | 48.0 | 9.0 | 16.7 | 71.0 | 55.0 | 18.0 | 25.0 | NA | 1.0 | 9.0 | 3.0 |
| Gao Y | China | 43 | 60.5 | 34.9 | NA | 45.0 | 30.2 | 16.3 | 69.8 | NA | 18.6 | NA | NA |
| Gayam V | America | 408 | 56.6 | 28.8 | 67.6 | 67.0 | 66.4 | 43.2 | NA | NA | 10.6 | 16.9 | NA |
| Ghweil AA | Egypt | 66 | 72.7 | 45.5 | 3.0 | NA | 16.7 | 16.7 | NA | NA | NA | NA | NA |
| Grasselli G | Italy | 3988 | 79.9 | NA | 48.3 | 63.0 | 42.1 | 12.9 | NA | NA | 2.3 | 2.2 | 8.3 |
| Gregoriano C | Switzerlan | 99 | 63.0 | 35.4 | 19.0 | 67.0 | 57.0 | 22.0 | NA | NA | 7.0 | 28.0 | 11.0 |
| Guan WJ | China | 1590 | 57.3 | 16.0 | 3.1 | 48.9 | 20.9 | 8.2 | 3.7 | NA | 1.5 | 1.3 | 1.1 |
| Guo T | China | 187 | 48.7 | NA | 23.0 | 58.5 | 32.6 | 15.0 | NA | 27.8 | 2.1 | 3.2 | 7.0 |
| Guo W | China | 174 | 43.7 | NA | 5.2 | 59.0 | 24.7 | 21.2 | 18.4 | NA | NA | 7.5 | 4.6 |
| Gupta S | America | 2215 | 64.8 | NA | 35.4 | 60.5 | 59.7 | 38.9 | NA | NA | 7.8 | 12.6 | 5.1 |
| Halvatsiotis P | Greece | 90 | 80.0 | NA | 28.9 | 65.5 | 50.0 | 18.9 | 21.1 | NA | 8.9 | 4.4 | 7.8 |
| Hu H | China | 105 | 50.9 | NA | 18.1 | 60.8 | 26.7 | 3.8 | 5.7 | NA | 11.4 | NA | 5.7 |
| Hu L | China | 323 | 51.4 | 53.3 | 10.8 | 61.0 | 32.5 | 14.6 | 12.7 | 7.4 | 1.9 | 2.2 | 1.5 |
| Huang H | China | 64 | 57.8 | 32.8 | 3.1 | 47.8 | 21.9 | 9.4 | NA | NA | NA | 4.8 | 4.8 |
| Huang R | China | 202 | 57.4 | 11.4 | 0.0 | 44.0 | 14.4 | 9.4 | 2.5 | NA | 3.5 | NA | 1.0 |
| Huang S | China | 310 | 56.1 | 50.0 | 18.7 | 62.0 | 36.5 | 15.5 | 6.1 | NA | NA | NA | 0.3 |
| Iaccarino G | Italy | 1591 | 64.0 | NA | 11.8 | 66.5 | 54.9 | 16.9 | NA | NA | 7.7 | 5.5 | NA |
| Inciardi RM | Italy | 99 | 81.0 | 19.0 | 26.0 | 67.0 | 64.0 | 31.0 | NA | NA | 9.0 | 15.0 | 18.0 |
| Itelman E | Israel | 162 | 64.8 | 16.0 | 3.1 | 52.0 | 30.2 | 18.5 | NA | NA | 1.2 | 1.2 | NA |
| Jang JG | Korea | 110 | 43.6 | 20.9 | 7.3 | 56.9 | 33.6 | 26.4 | 9.1 | NA | 3.6 | NA | 5.5 |
| Javanian M | Iran | 100 | 51.0 | NA | 19.0 | 60.1 | 32.0 | 37.0 | 20.0 | 14.0 | 12.0 | 12.0 | 4.0 |
| Ji W | Korea | 7341 | 40.5 | 13.0 | NA | 47.1 | 22.2 | 14.2 | NA | NA | NA | NA | 4.6 |
| Kalyanaraman | America | 6284 | 62.0 | NA | 28.0 | 61.0 | 37.0 | 33.0 | 24 | NA | 4 | 11 | 10.0 |
| Kim MK | Korea | 1082 | 35.5 | 14.7 | 7.9 | NA | 37.2 | 21.7 | 6.8 | NA | 6.7 | 3.0 | 5.5 |
| Lee JY | Korea | 694 | 24.8 | 19.7 | 2.6 | 52.1 | 18.9 | 11.7 | NA | NA | 0.6 | 0.7 | 3.3 |
| Lee JY (2) | Korea | 98 | 44.9 | NA | 28.4 | 72.0 | 52.0 | 27.6 | 16.3 | NA | 8.2 | 6.1 | 11.2 |
| Li J | China | 362 | 52.2 | 47.8 | 21.3 | 66.0 | NA | 35.1 | NA | NA | 5 | 9.7 | 3 |
| Li J (2) | China | 596 | 47.0 | 6.9 | 9.1 | 58.0 | NA | 13.1 | 36.1 | 21.1 | 0.7 | 1.8 | 4.5 |
| Li J (3) | China | 74 | 66.0 | 100.0 | 18.9 | 59.5 | 47.3 | 18.9 | NA | NA | NA | NA | 2.7 |
| Li K | China | 102 | 58.0 | NA | 14、7 | 57.0 | 30.0 | 15.0 | 4.0 | NA | 2.0 | NA | 5.0 |
| Li KH | China | 83 | 53.0 | 30.1 | NA | 45.5 | 6.0 | 7.8 | 0.0 | NA | 6.0 | NA | NA |
| Li Q | China | 325 | 51.4 | 8.0 | 0.9 | 51.0 | 24.0 | 9.2 | 5.5 | 9.2 | 1.2 | 1.2 | 0.9 |
| Li T | China | 312 | 59.9 | 33.7 | 6.7 | 69.2 | 57.1 | 38.8 | 29.8 | 33.0 | 8.7 | 3.2 | 3.9 |
| Liu J | China | 1190 | 53.4 | 3.9 | 13.2 | 57.0 | 26.1 | 12.2 | 7.3 | NA | 1.9 | 2.6 | 2.9 |

| | | | | | | | | | | | | | |
|---|---|---|---|---|---|---|---|---|---|---|---|---|---|
| Liu JY | China | 61 | 50.8 | 27.9 | NA | 40.0 | 19.7 | 8.2 | 1.6 | NA | 8.2 | NA | NA |
| Liu L | China | 51 | 62.7 | 13.7 | NA | 45.0 | 7.8 | 7.8 | NA | NA | NA | NA | NA |
| Lv Z | China | 354 | 49.4 | 43.8 | 3.1 | 62.0 | 20.9 | 9.9 | NA | NA | 1.7 | NA | 0.6 |
| Ma KL | China | 84 | 57.1 | 23.8 | NA | 48.0 | 14.3 | 11.9 | 6.0 | 42.9 | 6.0 | 1.2 | 1.2 |
| Mao L | China | 214 | 40.7 | 41.1 | NA | 52.7 | 23.8 | 14.0 | 7.0 | NA | NA | 2.8 | 6.1 |
| Mehra MR | Asia, Europe, and America | 8910 | 59.9 | NA | 5.8 | NA | 26.3 | 14.3 | NA | NA | 2.3 | NA | NA |
| Mikami T | America | 2820 | 48.9 | NA | 28.6 | NA | 33.0 | 23.3 | NA | NA | 3.8 | 11.2 | 6.9 |
| Nowak B | Poland | 169 | 51.5 | 16.0 | 27.2 | 63.7 | 47.3 | 18.9 | 34.3 | NA | 13.0 | 20.7 | 20.7 |
| Pan F | China | 124 | 68.5 | 73.4 | 71.8 | 68.0 | 50.0 | 20.2 | 15.3 | NA | 8.9 | NA | NA |
| Pellaud C | Switzerlan | 196 | 61.0 | 25.0 | 16.8 | 70.0 | 60.0 | 27.0 | NA | NA | 8.0 | NA | 8.0 |
| Qin C | China | 452 | 52.0 | 63.3 | NA | 58.0 | 29.5 | 16.4 | 5.9 | NA | 2.6 | 2.2 | 3.1 |
| Ruan Q | China | 150 | 68.0 | NA | 45.3 | NA | 34.7 | 16.7 | 8.7 | NA | 3.0 | 3.0 | 3.0 |
| Schalekamp S | Netherlan | 356 | 67.0 | 47.0 | 27.2 | 69.0 | 37.4 | 18.5 | 27.8 | NA | 19.4 | 14.3 | NA |
| Shah P | America | 522 | 41.8 | 23.6 | 17.6 | 63.0 | 79.7 | 42.3 | NA | NA | 9.0 | 14.9 | 9.2 |
| Shahriarirad R | Iran | 113 | 62.8 | 9.7 | 8.0 | 53.8 | 19.5 | 14.2 | 14.2 | NA | 8.0 | 5.3 | 0.9 |
| Shi S | China | 416 | 49.3 | NA | 13.7 | 64.0 | 30.5 | 14.4 | NA | 19.7 | 2.9 | 3.4 | 2.2 |
| Shi S (2) | China | 671 | 48.0 | 100.0 | 9.2 | 63.0 | 29.7 | 14.5 | NA | NA | 3.4 | 4.2 | 3.4 |
| Shi Y | China | 487 | 53.2 | 10.1 | NA | 46.0 | 20.3 | 6.0 | 2.3 | NA | NA | 1.4 | 1.0 |
| Sun H | China | 244 | 51.4 | NA | 49.6 | NA | 56.6 | 20.9 | NA | NA | NA | NA | NA |
| Tabata S | Japan | 71 | 55.0 | 39.4 | NA | 67.0 | NA | 3.0 | 31.0 | NA | NA | NA | 4.0 |
| Tian J | China | 751 | 50.0 | 42.0 | 14.0 | 64.0 | 39.0 | 26.0 | NA | NA | 1.0 | 3.0 | 30.9 |
| Wan S | China | 135 | 53.3 | 29.6 | 0.7 | 47.0 | 9.6 | 8.9 | 5.2 | 7.4 | 0.7 | NA | 3.0 |
| Wang B | China | 483 | 45.1 | 12.8 | NA | 48.4 | 7.0 | 5.0 | NA | NA | NA | NA | 1.0 |
| Wang D | China | 107 | 53.3 | NA | 17.8 | 51.0 | 24.3 | 10.3 | 12.1 | 11.2 | 2.8 | 2.8 | NA |
| Wang K | China | 548 | 50.9 | NA | 14.2 | 60.0 | 30.3 | 15.1 | NA | 21.7 | 3.1 | 1.8 | 4.4 |
| Wang K (2) | China | 296 | 47.3 | NA | 6.4 | 47.3 | 14.2 | 10.1 | NA | NA | 0.7 | 1.7 | 0.3 |
| Wang W | China | 421 | 50.8 | 14.0 | NA | 52.0 | 10.5 | 3.1 | NA | NA | 4.3 | NA | 1.0 |
| Wang Y | China | 334 | 52.0 | NA | 39.0 | 64.0 | 41.0 | 18.6 | 11.6 | 32.3 | 4.7 | NA | NA |
| Wang YF | China | 110 | 43.6 | 34.5 | 65.3 | NA | 20.9 | 13.7 | NA | NA | 5.5 | NA | NA |
| Wang YP | China | 275 | 46.5 | 16.4 | 0.0 | 49.0 | 19.6 | 6.2 | NA | NA | NA | 1.5 | 0.7 |
| Wei JF | China | 101 | 53.5 | 36.6 | 3.0 | 49.0 | 21.0 | 13.9 | NA | 5.9 | 1.0 | NA | NA |
| Wei Y | China | 276 | 56.2 | 5.1 | 1.8 | 51.0 | 17.0 | 5.1 | NA | NA | 2.5 | NA | 1.1 |
| Wu C | China | 201 | 63.7 | 41.7 | 21.9 | 51.0 | 19.4 | 10.9 | 4.0 | NA | 2.5 | 1.0 | 0.5 |
| Xie H | China | 79 | 55.7 | 35.4 | NA | 60.0 | 17.7 | 10.1 | NA | NA | NA | NA | NA |

| | | | | | | | | | | | | | |
|---|---|---|---|---|---|---|---|---|---|---|---|---|---|
| Xie Y | China | 62 | 43.5 | 38.7 | 3.2 | 66.0 | 38.7 | 21.0 | 53.2 | NA | NA | NA | NA |
| Xu J | China | 239 | 59.8 | 100.0 | 61.5 | 62.5 | 43.9 | 5.4 | 14.6 | NA | 5.0 | NA | 18.4 |
| Xu PP | China | 703 | 54.0 | 15.2 | 4.7 | 46.1 | 17.0 | 9.0 | 5.0 | NA | 2.0 | 1.0 | 1.0 |
| Yang Q | China | 136 | 48.5 | 33.2 | 16.9 | 56.0 | 27.1 | 14.7 | 6.6 | 8.1 | NA | 2.9 | 2.9 |
| Yang X | China | 52 | 67.0 | 100.0 | 61.5 | 59.7 | NA | 0.2 | 10.0 | 23.0 | 8.0 | NA | 4.0 |
| Yao Q | China | 108 | 39.8 | 12.0 | 11.8 | 52.0 | 14.8 | 4.6 | 3.7 | 7.2 | 2.8 | NA | 1.9 |
| Ye C | China | 856 | 51.3 | 18.0 | 0.1 | 46.0 | 16.6 | 7.5 | 1.5 | NA | NA | 0.8 | 0.9 |
| Yu C | China | 1663 | 50.4 | 52.0 | 10.2 | 64.0 | 20.9 | 14.7 | NA | NA | 3.7 | 1.9 | 1.1 |
| Yu C (2) | China | 1464 | 50.3 | NA | 14.5 | 64.0 | 20.9 | 14.4 | NA | NA | 3.4 | 1.8 | 1.2 |
| Yu X | China | 333 | 51.7 | 7.8 | 0.6 | 50.0 | 19.2 | 8.4 | 7.2 | NA | NA | NA | NA |
| Yuan M | China | 27 | 45.0 | NA | 37.0 | 60.0 | 19.0 | 22.0 | 11.0 | NA | NA | NA | 4.0 |
| Zhang F | China | 48 | 68.7 | 45.8 | 35.4 | 70.6 | 66.7 | 20.8 | NA | 27.1 | NA | 10.4 | NA |
| Zhang GQ | China | 221 | 48.9 | 24.9 | 5.4 | 55.0 | 24.4 | 10.0 | 10.0 | 7.7 | 2.7 | 2.7 | 4.1 |
| Zhang JJ | China | 140 | 50.7 | 41.4 | NA | 57.0 | 30.0 | 12.1 | NA | 5.0 | 1.4 | 1.4 | NA |
| Zhang SY | China | 788 | 51.6 | 9.9 | 0.0 | NA | 16.0 | 7.2 | 1.4 | NA | 0.4 | 0.9 | 0.8 |
| Zhang XY | China | 91 | 53.8 | 33.0 | 2.2 | 46.0 | 19.8 | 3.3 | NA | 15.4 | 1.0 | 1.0 | 3.3 |
| Zhang Y | China | 258 | 53.5 | 66.3 | 5.8 | 64.0 | 38.0 | 24.4 | 15.1 | 7.4 | 3.5 | 3.5 | 4.7 |
| Zhao J | China | 29 | 48.3 | 72.4 | 3.4 | 56.0 | NA | 24.1 | 34.5 | NA | NA | NA | NA |
| Zhao W | China | 77 | 44.2 | 26.0 | NA | 52.0 | 20.8 | 7.8 | 11.7 | NA | 7.8 | 6.5 | 5.2 |
| Zheng F | China | 161 | 49.7 | 18.6 | NA | 45.0 | 13.7 | 4.3 | NA | NA | 3.7 | NA | NA |
| Zheng S | China | 96 | 60.0 | 77.1 | NA | 55.0 | 36.0 | 11.0 | 7.0 | NA | 4.0 | 1.0 | 1.0 |
| Zhu L | China | 7337 | 47.4 | 8.5 | 3.4 | 54.0 | 24.0 | 13.0 | NA | 3.5 | 0.8 | 1.8 | NA |
| Zhu Z | China | 127 | 35.4 | 12.6 | NA | 50.9 | 24.4 | 7.9 | 4.7 | NA | 4.7 | NA | 3.9 |

CVD: cardiovascular disease; COPD： chronic obstractive pulmonary disease; CKD: chronic kidney disease; NA: not available.

sTable 2: The pooled outcome in patients with comorbidities or cardiac injury.

| Co-morbidity | Effect size | | | Publication bias |
|---|---|---|---|---|
| | OR | 95% CI | P-value | P (Egger's test) |
| Severe COVID-19 | | | | |
| Hypertension | 2.565 | 2.117-3.108 | 0.000 | 0.360 |
| DM | 2.542 | 1.893-3.414 | 0.000 | 0.001 |
| CVD | 3.863 | 2.704-5.518 | 0.000 | 0.411 |
| COPO | 2.710 | 1.984-3.702 | 0.000 | 0.698 |
| CKD | 2.196 | 1.268-3.802 | 0.005 | 0.228 |
| Cancer | 2.416 | 1.815-3.217 | 0.000 | 0.552 |
| Cardiac injury | 6.568 | 3.70411.646 | 0.000 | 1.000 |
| Mortality | | | | |
| Hypertension | 2.504 | 2.017-3.109 | 0.000 | 0.542 |
| DM | 2.087 | 1.803-2.416 | 0.000 | 0.991 |
| CVD | 2.654 | 1.869-3.769 | 0.000 | 1.000 |
| COPO | 2.478 | 2.047-2.999 | 0.000 | 0.840 |
| CKD | 3.072 | 2.434-3.878 | 0.000 | 0.66 |
| Cancer | 1.900 | 1.572-2.297 | 0.000 | 0.247 |
| Cardiac injury | 16.959 | 7.892-36.441 | 0.000 | 1.000 |
